# Hepatitis B virus genotypes and evolutionary profiles from blood donors from the northwest region of China

**DOI:** 10.1186/1743-422X-6-199

**Published:** 2009-11-17

**Authors:** Xing-bin Hu, Qiao-hong Yue, Xian-qing Zhang, Xue-qing Xu, Yin Wen, Yao-zhen Chen, Xiao-dong Cheng, Liu Yang, Shi-jie Mu

**Affiliations:** 1 Department of Blood Transfusion, Xijing Hospital, the Fourth Military Medical University, 17th Changlexi Road, Xi'an 710032, PR China; 2 Department of Clinic Molecular Research Center & Clinic Diagnostic Laboratory, Xijing Hospital, Fourth Military Medical University, 17th Changlexi Road, Xi'an 710032, PR China; 3 Department of Molecular Genetics, Third Military Medical University, Gaotanyan, Chongqing, 40038, PR China; 4 Department of Electron Microscope, Centralab, Fourth Military Medical University, 15th Changlexi Road, Xi'an 710032, PR China

## Abstract

Hepatitis B virus (HBV) is prevalent in China and screening of blood donors is mandatory. Up to now, ELISA has been universally used by the China blood bank. However, this strategy has sometimes failed due to the high frequency of nucleoside acid mutations. Understanding HBV evolution and strain diversity could help devise a better screening system for blood donors. However, this kind of information in China, especially in the northwest region, is lacking. In the present study, serological markers and the HBV DNA load of 11 samples from blood donor candidates from northwest China were determined. The HBV strains were most clustered into B and C genotypes and could not be clustered into similar types from reference sequences. Subsequent testing showed liver function impairment and increasing virus load in the positive donors. This HBV evolutionary data for China will allow for better ELISA and NAT screening efficiency in the blood bank of China, especially in the northwest region.

## Introduction

Hepatitis B virus (HBV) poses a great threat to humans, with serious consequences including liver cirrhosis, hepatocellular carcinoma and polyarteritis nodosa [1]. This infection is prevalent in Asia, Africa, Southern Europe and Latin America [2]. Roughly 2 billion people, one-third of the world's population, have serological evidence of past or ongoing infection with HBV. Approximately 5-10% of infected adults and 80-90% of children become chronic carriers of HBV [3,4]. China has been heavily affected over a considerable period of time; consequently about 10% of the population are carriers or sufferers [5].

Because of the high prevalence of HBV, the blood bank of China must screen donors for HBV infection [6]. All samples from blood donors are tested for HBV surface antigen (HBsAg) and alanine amino transferase (ALT). HBsAg is currently identified by ELISA, and ALT is tested for using dynamic enzyme methods. Undoubtedly, such screening is instrumental in reducing the risk of HBV transmission through blood transfusion [7]. However, as mutations can occur in different viral stains, ELISA occasionally fails to detect HBV-infected donors [8-11].

DNA tests have revealed that HBV strains from blood donors vary in different geographical areas. Occult HBV infection, which threatens the safety of blood transfusion, is linked at least in part to the genetic distance of the viral strains [12]. Nucleic acid testing (NAT) for HBV in a large number of blood donors has identified HBV DNA-positive but HBsAg-negative donors, providing a unique opportunity to investigate HBV infection in more detail [13,14].

Although the DNA test is superior to ELISA and can overcome some of its disadvantages, in China the higher cost and imperfect protocols have prevented widespread use. Consequently, until now, ELISA has remained the major testing method in China. To improve HBsAg testing, it is necessary to probe HBV virus evolution, because evolutionary analysis will help to promote ELISA innovations [15]. However, data about HBV evolution in Chinese donors, especially in the northwest region of China, is not yet available, creating a hurdle for the development of more efficient testing.

Here we reported that 11 HBV strains from northwest China blood donor candidates were mostly clustered into B and C genotypes. These pathogens, which appear to have developed from a common parent, could not be clustered into similar genotypes from the reference sequences. This points to a high mutation frequency of HBV. Follow-up testing showed liver function impairment and increased virus load in these positive donors. Our research has supplied HBV evolution data and will pave the way for improving ELISA and NAT screening in the blood bank of China.

## Materials and methods

### Blood donor recruitment and sample collection

120 donors, negative for anti-HCV and anti-HIV antibodies, were analyzed. All recruited donors were unremunerated volunteers from either urban or rural areas. They were medically assessed and via a questionnaire denied any known risk factors for viral infection. Donors found to be HBV carriers were also asked to give follow-up blood samples for further study. The study was approved by the Ethics Committee of Fourth Military Medical University and written informed consent was obtained from the participants.

### HBV serological marker determination

Testing for HBV serological markers, including HBs, anti-HBs, anti-HBe, HBe and anti-HBs, were performed by ELISA using an automatic enzyme detection system (Tecan, Swiss) and a commercial kit (InTec Products, China) according to the manufacturers' protocols. For the quantitative detection of the markers, serum from blood donors was applied to AXSYM MEIA (Abbott Diagnostics, Germany). To measure the ALT level, serum was separated and run through an automatic biochemistry analyzer (Hitachi, Japan) using Kit (Shanghai Fousun Long March Medical Scince.Co.Ltd. China).

### 2.3 DNA analysis

NAT was adapted for the current study as previously described [16]. Briefly, 120 donor blood samples were divided into 10 pools with 12 samples each pool. DNA from the blood samples was extracted according the manufacturer's protocol (Qiagen, Germany) and mixed [16]. Real-time PCR was used to detect HBV in each pool, following the manufacturer's instructions (Qiagen, Germany). If a positive reaction was observed, the pool was divided into 6 samples and real-time PCR was repeated. If there was a second positive test, each individual sample was tested. After that, quantitive PCR was employed for to quantify viral load.

As Katsoulidou *et al*. described [17], positive samples were genotyped using nest-PCR. Briefly, the first-round PCR primers (outer primer pairs) and second-round PCR primers (inner primer pairs) were designed on the basis of the conserved nature of nucleotide sequences in the regions of the pre-S1 through S genes. At the end, agar electrophoresis was employed to discern genotype.

11 HBV DNA reactive samples were randomly picked out (hereafter referred to as donors 1 to 11). From these samples, HBV DNA was extracted from 1.0 mL of serum using a kit (Qiagen GmbH, Germany), according to the manufacturer's instructions. Then, sequence analysis, beginning from the S region of HBV genome, was performed by an external company (Sunbiotech. Ltd China) using an ABI sequencing system.

### Phylogenetic analysis

HBV genome phylogenetic analysis was performed by multiple sequence alignment using the ClustalW v1.83 program [18]. For this purpose, HBV sequences from the donors and reference sequences from the GenBank database http://www.ncbi.nih.gov were aligned.

## Results

### NAT screening of 11 HBV-infected donors

**Figure 1:**
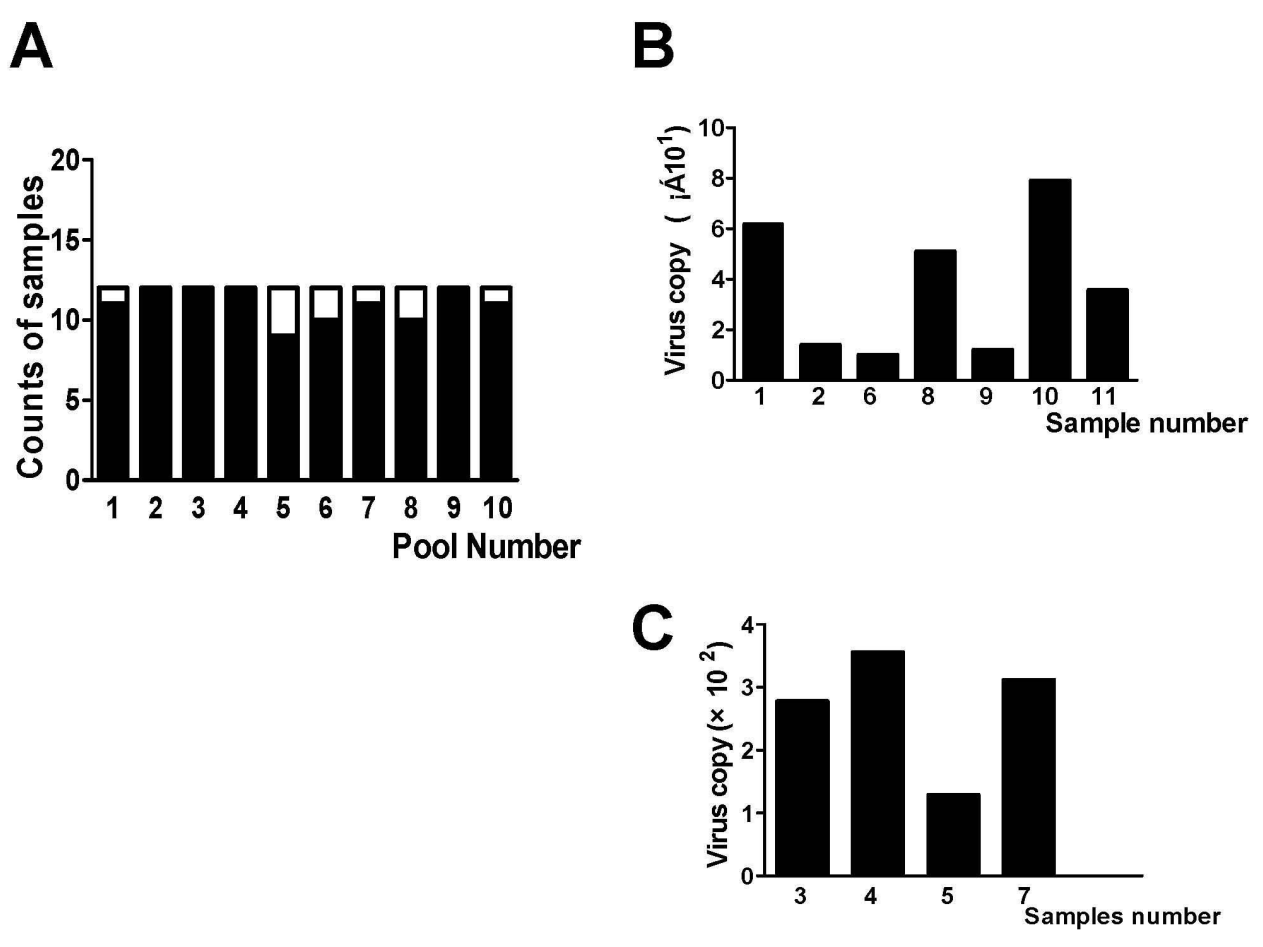
**NAT screening of 11 reactive samples from 120 blood donors**. To screen the HBV infected donors, NAT was employed. 120 donor blood samples were divided into 10 pools with 12 samples in each pool. Real-time PCR was used on each pool. If a positive reaction was observed, the pool was narrowed until a single reactive sample was detected. Quantitive PCR was then performed against positive samples. A, reactive sample counts of each pool in the first round of detection; blank represents HBV-reactive sample counts and black is the total sample counts in the pool; B, lower HBV DNA copies of reactive samples in the NAT-reactive samples; C higher HBV DNA copies of reactive samples in the NAT-reactive samples.

To screen the HBV-infected donors, NAT was employed based on real-time PCR. In the first round of screening, there were 6 positive pools (Fig. 1A, 60%). A single positive HBV sample was eventually identified by repeat real-time PCR. There were 12 reactive blood donors, representing about 9% of all the recruited donors.

Next, HBV DNA load in the positive donors was measured. As shown in Figures 1B and 1C, donors 3, 4, 5 and 7 had several hundred virus copies, while donors 1,2,6,8,9,10 and 11 had lower virus loads.

The serological and personal data of the HBV DNA-reactive donors are noted in Tables 1 and 2. Consistent with virus load, donors 3, 4, 5 and 7 had higher ALT levels (Table 1 and Fig. 1C), which were all beyond the upper limit (20 IU.L^-1^) for blood donors formulated by China. On the other hand, serological marker analysis of HBV in the samples showed that donors 3 and 4 were more contagiousness, as they were HBsAg, HBeAg and anti-HBc positive (Table 2). Although HBsAg in donor 6 was negative (Table 2), HBV DNA testing proved there were few virus copies (Fig. 1B).

**Table 1:** Data of blood donors with positive HBsAg reaction

**NO**.	Year	Femal/Male	ALT(U.L-^1^)
1	26	M	17
2	44	F	12
3	32	F	39
4	22	M	49
5	39	M	29
6	50	M	19
7	25	M	47
8	41	F	15
9	21	F	19
10	43	M	20
11	35	F	21

**Table 2:** Serological markers of donors bearing HBV

**NO**.	HBs	Anti-HBs	HBe	Anti-HBe	Anti-HBc
1	+	+	-	-	+
2	+	-	-	+	+
3	+	-	+	-	+
4	+	-	+	-	+
5	+	-	+	-	-
6	-	-	-	-	-
7	+	+	-	-	+
8	+	-	-	+	+
9	+	+	-	-	+
10	+	-	-	-	+
11	+	-	-	+	-

**Total**	10	3	3	3	8

### The major HBV strains in local donors were B and C genotypes

**Figure 2:**
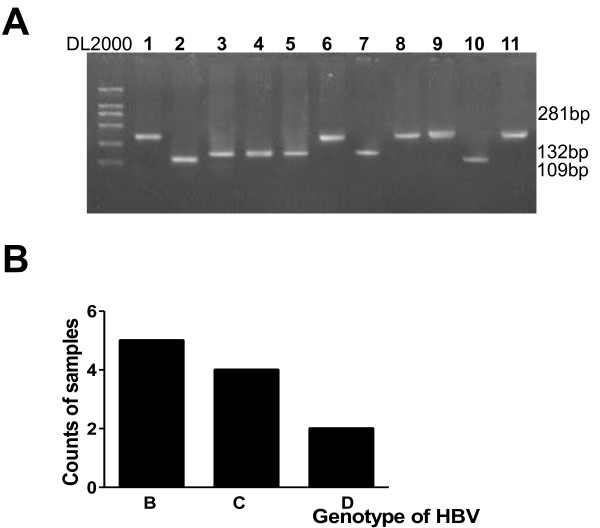
**Genotyping of HBV reactive blood donors**. All positive samples from the blood donors were subjected to DNA extraction and then genotyping by nest-PCR. A, gel electrophoresis of PCR products after nest-PCR in genotype analysis (data represents one of three independent experiments); B, sample counts of each genotype according A.

HBV strains vary in different regions and different strains may contribute to ELISA test failure. To further discern the HBV type in the infected samples, all 11 positive samples underwent HBV genotyping by PCR (Fig. 2A). 9 virus strains, from approximately 81% of all the positive samples, belonged to the B or C group, but 2 D genotype strains (19%) were also observed (Fig. 2B). Furthermore, the HBV DNA load in cases with the C subtype was higher than that in the B or D genotypes (Fig. 1B and 1C).

### HBV strains from local donors evolved from common parents

**Figure 3:**
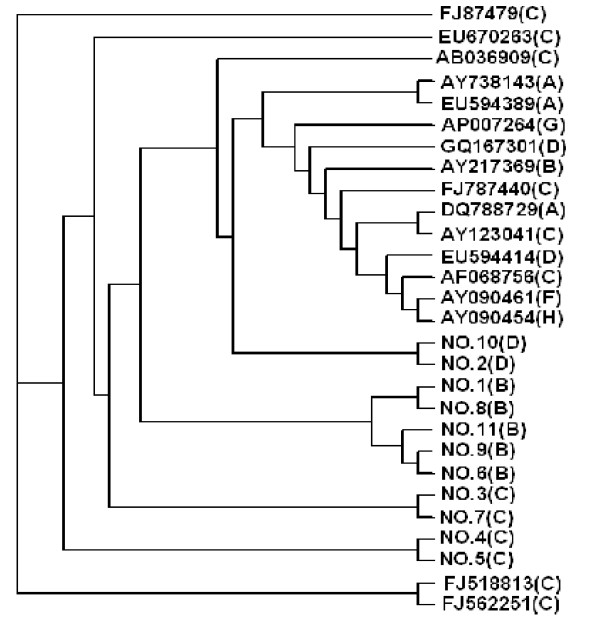
**Phylogenetic analysis of HBV strains from the blood donors**. HBV genome sequencing was carried out. Some available sequences from the GenBank database were used to construct the tree with the Clustal W v1.83 program. Figures in the lower part of the tree are the blood donors' numbers; characters in the other part of the tree are serial numbers in GeneBank; characters in the right bracket refer to HBV genotype.

HBV has evolved in recent years. This evolution has resulted in blood transfusion transmission because of ELISA test failures. Since most of the HBV strains in the current study belonged to the B or C genotypes, we further sequenced the HBV-DNA positive samples to make a phylogenetic appraisal. Fortunately, all the strains were successfully sequenced. Then, a phylogenetic tree was made, joined by reference sequence from GeneBank using Clustal W 1.83 software. According to the tree, the 2 donor samples belonging to the D genotypes (donors 2 and 10) were highly homogenic, while the 4 C strains (donors 3, 4, 5 and 7) came from a common 'parent' (Fig. 3). With the 5 B strains, the situation was more complex. Although they derived from the same root, two evolutionary directions were identified. As shown in Figure 3, the virus strain from donors 1 and 8 was clustered, while the strain from donors 6, 9 and 11 belonged to another group.

On the whole, however, the strains could not be clustered into a similar subtype using reference sequences, which proves the high mutation rate of HBV.

### Infection of HBV-positive donors worsened in the following 3 years

To monitor HBV infection after the preliminary analysis, we tracked the positive blood donors in the following years. Two years later, donor 6, who was negative reaction in the preliminary serological test, became reactive against HBsAg, while the other donors displayed increased positive markers of infection (Table 3). Repeat ALT testing showed that the liver function of these donors was, at least partly, impaired (Table 4). We noted that the level of ALT in donor 3 decreased because she received anti-viral therapy (Table 4). Virus load was serially quantitated by real-time PCR. As shown in Table 5, the number of virus copies increased in all reactive donors, except donor 3 (who was being treated). Once again, several hundred HBV copies were detected in donor 6.

**Table 3:** Quantitive levels of serological markers of HBV in reactive donors two years later

**NO**.	HBs(nq.mL^-1^)	Anti-HBs (mIU.mL^-1^)	HBe (ncuq.mL^-1^)	Anti-HBe (ncuq.mL^-1^)	Anti-HBc (ncuq.mL^-1^)
donor 1	2.8	32.1	0.02	6.2	11.8
donor 2	3.1	41.0	0.08	5.9	8.3
donor 3	15.2	192.5	2.5	121.8	31.2
donor 4	19.8	288.1	10.1	117.4	15.7
donor 5	62.9	162.3	8.4	125.9	12.1
donor 6	1.9	24.2	0.01	2.7	0.9
donor 7	7.5	69.8	8.7	18.1	11.9
donor 8	12.7	32.2	0.09	7.3	7.1
donor 9	13	33.9	0.01	6.9	5.9
donor10	2.6	42.3	0.02	8.5	6.7
donor11	12.5	51.7	0.07	6.6	6.8

ncu: national clinical unit.

**Table 4:** ALT levels in the consecutive test of HBV reactive donors

Time(month)	ALT(U.L-^1^)											
	
	3	6	9	12	15	18	21	24	27	30	33	36
donor 1	18	20	19	26	30	33	39	40	49	56	60	63
donor 2	11	15	18	24	32	43	49	51	59	67	N	N
donor 3	35	50	57	68	78	90	102	110	123	151	130	107
donor 4	55	54	55	62	68	65	71	75	80	86	90	96
donor 5	32	34	39	40	40	47	48	46	50	56	57	59
donor 6	20	20	19	26	28	23	39	40	45	46	N	48
donor 7	47	53	57	56	58	60	61	59	70	76	80	87
donor 8	12	14	15	12	18	25	21	25	20	26	32	34
donor 9	14	14	17	20	19	21	22	25	20	21	N	N
donor 10	21	20	29	26	28	23	29	30	N	40	41	40
donor 11	26	25	29	27	28	33	29	31	38	41	35	44

N: no test.

**Table 5:** Virus load in the following test of HBV reactive donors

Time(month)	Virus Copy					
	
	6	12	18	24	30	36
donor 1	6.1E1	7.9 E1	8.8 E1	10 E2	15 E3	21 E3
donor 2	2.1 E1	5.3 E1	6.7 E1	8.2 E2	11.8 E1	N
donor 3	2.8 E2	5.4 E2	2.3 E3	3.5 E3	1.2 E3	2.1 E3
donor 4	3.9 E2	8.3 E2	4.3 E3	7.5 E3	0.3 E4	32.0E4
donor 5	1.6 E2	44 E2	1.3 E3	4.5 E3	3.2 E4	4.9 E4
donor 6	1.2 E1	3.9 E1	6.2 E1	9.8 E2	11 E2	1.7 E3
donor 7	3.2 E2	24.1 E2	86.7 E2	2.5 E3	8.2 E3	14.1 E3
donor 8	5.7 E1	6.3 E1	7.5 E1	9.4 E2	10.1 E2	14.9 E3
donor 9	1.3 E1	4.2 E1	5.9 E1	7.2 E2	7.8 E2	9.3 E3
donor 10	8.6 E1	9.9 E1	16.7 E1	2.5 E2	5.3 E2	14.6 E2
donor 11	4.5 E1	5.6 E1	16.8 E1	4.1 E2	12.3 E2	25.3 E2

N: no test. E1:10^1^; E2:10^2^;E3:10^3^;E4:10^4^.

## Discussion

NAT has been globally adopted in blood banks to detect infectious pathogens, especially in developed nations [16]. With a proper pool size, it can detect several virus copies in a sample. In this way, the testing window period of pathogens, which is one of the most important risk factor in transfusion medicine, can be overcome. However, due to lower numbers of virus copies during early stages of infection, NAT sometimes produces false negative results. Consequently, pool size becomes a key factor in interpreting NAT. In the current study, our NAT system could detect 10 copies of HBV. This sensitivity was enough to detect the virus in a 6-sample pool.

Other disadvantages have prevented the wider uptake of NAT [19]. One complex issue is how to determine the appropriate blood donor pool size [20,21]. We screened 11 reactive samples from 120 blood donors using NAT. The samples were divided into 10 pools and each pool contained 12 samples. In effect, 3 real-time PCRs were run before the single reactive sample was found. If we made larger pools, perhaps more real-time PCRs would have been performed, given the high prevalence of HBV in China. We found that the test results from NAT were almost perfectly consistent with ELISA testing (9.16% *V.S *8.33%, *P *> 0.05), although the latter failed in donor 6 due to lower HBV virus copy numbers. We therefore agree with earlier authors that ELISA can be used as the first round test and NAT in the second round analysis [22].

In 1988, Okamoto *et al*. categorized HBV into A, B, C and D types according the genome sequence diversity [23]. There are now 8 known genotypes of HBV, from A to H, with a genome difference greater than 8% [24,25]. Genotypes A, B, C and D are all observed in China. Consistent with other reports, we confirmed that the B and C genotypes are the most common in China [26]. Genotype of HBV is significant to prognosis and test strategy [27]. We found that blood donors with the C genotype had higher virus loads and more serious liver impairment in the following years, which is consistent with the findings of other researchers [28].

According to our serological data, the 11 positive samples could be divided into two major groups: 6 subjects (54.5%) were anti-HBc positive without detectable antibodies to surface antigen, whereas 3 (27.2%) were positive for both anti-HBc and anti-HBs. These results indicate that occult HBV infection can occur in blood donors [29,30]. It has been reported that occult HBV infection without anti-HBs is more dangerous because cases of transmission by donations carrying anti-HBc without anti-HBs have been documented, while no evidence of transmission has been found when donors were both anti-HBc and anti-HBs reactive [31,32].

Phylogenetic analysis is a method commonly used to trace virus evolution [33]. With HBV, an evolutionary tree can be drawn from a partial sequence or the whole genome [34]. We made a genome tree and, surprisingly, found that the strains in our region could not be clustered into similar types using reference sequences. The reason may be the high mutation rate of HBV, for which there is considerable supporting evidence [35,36]. However, virus subtypes B or C, which were found in the present study, are clues suggesting different evolutionary roots. We likewise did not detect recombinant HBV strains, which have occasionally been reported as intertypes [37,38]. Recombinant HBV strains, if present, might contribute to the diverse phylogenetic profile in our region. On the other hand, the virus strains we detected that were of the same type had clearly developed from the same progenitors, which suggested that local HBV evolution had specific characteristics. This phenomenon is relevant for both ELISA and NAT improvement.

In summary, the 11 HBV strains from northwest China blood donor candidates which we identified were mostly clustered into B and C genotypes. These organisms could not be clustered into similar types using reference sequences. Follow-up testing showed liver function impairment and increasing virus load in the positive donors. The study provides evolutionary data about HBV in China and could lead to improvements in ELISA and NAT screening efficiency in the blood bank of China.

## Competing interests

The authors declare that they have no competing interests.

## Authors' contributions

HXB carried out the donors secreen and drafted the manuscript. YQH participated in the sequencing. ZXQ performed NAT analysis. XXQ carried out molecular genetic studies. YW carried out genotyping. CYZ and CXD participated in the follow-up test of ALT. YL participated in the ELISA test analysis. MSJ partipated in the design of the study. All authors read and approved the final manuscript.
